# Supplementary material for: The use of health geography modeling to understand early dispersion of COVID-19 in São Paulo, Brazil
Source: PLoS One. 2021 Jan 7;16(1):e0245051. doi: 10.1371/journal.pone.0245051 (PMC7790416; doi:10.1371/journal.pone.0245051)
Supplement: Attachment — Submitted filename: Response_to_Reviewers.docx [file pone.0245051.s003.docx]

Botucatu, December 15^th^ 2020,

Response to reviewers,

We thank the reviewers for their careful reading and suggestions. Below, we answer the questions, clarifying points, and highlighting the changes made regarding the arisen points.

Reviewer #1: **The authors conducted a thorough review of the article.**

**However, regarding the mathematical model, figure 6 clearly demonstrates that the SEIR model, as simulated, is not able to adequately represent the evolution of the disease in the state (points in blue).**

**This is probably a result of the interventions adopted and that are not implemented in the model. One way to better model social distancing would be to make the beta value variable during the simulation by adjusting it based on the data (capturing the dynamics of social distancing). This would make the simulations more accurate. However, since the model does not contribute to the central hypothesis of the article, I suggest its removal.**

We thank the comment, and we agree entirely with it. Perform estimation for different betas throughout the time would diverge from the work goal and its scope; therefore, we opted for the removal of the mathematical model from the text.

Reviewer #2: **Authors have made majority of the changes as suggested and have provided appropriate explanations when applicable. Overall the presentation of the paper has improved compared to the previous submission and in a good shape for publication. Minor suggestions are listed below.**

**Abstract and introduction**

**Still the hypothesis is not clearly presented. It seems the authors hypothesized a 'diffusion' spread and eventually suggested that the hierarchy of the regions matters compared to the contiguity. I suggest to consider revision.**

We rewrote the last paragraph of the introduction to make it clear (lines 23-40). We also change the word "diffusion" to "dispersion" to avoid misunderstanding. We hope that it is clear now. In red, the changes are highlighted. "*Here, we discussed a detailed analysis of the spatial dispersion of COVID-19 in São Paulo State, Brazil, intending to provide real-time responses to support public health strategies. Using data since the first confirmed cases of COVID-19 in São Paulo State, we assess the importance of geographic space on the spread of the epidemic. We hypothesize that urban hierarchy is the main responsible for the disease spreading, and we identify the hotspots and the main routes of virus movement from the metropolis to the inner state. This premise is also supported by [8] where multivariate analyses showed that demographic density and high classification of regional relevance were associated with early introduction and high COVID-19 incidence and mortality rates. In work developed here, we cross-validate the confirmed cases with urban mobility, urban hierarchy, and land use at each spatial localization. The results highlight the importance of the main routes that cross São Paulo State and the regional airports on the introduction of the disease in the territory, just as the main municipalities that act as key centers of disease spreading to the inner state. Knowing in advance the path of COVID-19 dispersion can support decision-makers to optimize health service and plan strategies of quarantine measures. This approach can be done in other states of Brazil and other developing countries, observing local and regional differences of mobility and urban network.*"

In the abstract of the first revision version the sentence "*We hypothesize that urban hierarchy is the main responsible for the disease spreading, and we identify the hotspots and the main routes of virus movement from the metropolis to the inner state*" was already added.

The following reference was added:

[8] Fortaleza CMCB, Guimarães RB, de Almeida GB, Pronunciate M, Ferreira CP (2020). Taking the inner route: spatial and demographic factors affecting vulnerability to COVID-19 among 604 cities from inner São Paulo State, Brazil. Epidemiology and Infection 148, e118, 1–5. <https://doi.org/10.1017/> S095026882000134X

**Methods**

**1. SDE is simply a descriptive representation of the mean center and directionality of the cases at a given time. While authors have put good effort to explain SDE calculation, it is unclear why the three days were selected March 29th, April 8th, and April 18th. Please explain.**

We added the following sentence to justify the calendar days used (lines 121-130): "*Although the SARS-CoV-2 was introduced in São Paulo on March 25th, it took time to move from it to the inner municipalities because of the strong mitigations strategy adopted by São Paulo State to halting the disease's spread. The average time spent by the disease, since its introduction on the metropolis, to achieve the regional centers, the municipalities under major and minor influence, and the rural municipalities were respectively 22, 31, 34, and 55 days [8] (the classification of the municipalities follows the criteria established by the Brazilian Institute for Geography and Statistics (2017) [17]). Therefore, three calendar dates were chosen to cover the study period (from March 25th until April 18th): March 29th, April 8th, and April 18th, which are 10 days apart from each other.*"

**2. It is unclear how the airplanes (Fig 2) map was used in the analysis?**

Fig. 2 emphasizes the movement restrictions implemented during the social distancing reflected in the number of people traveling in São Paulo State, but also highlight that it took time to occur, and this delay helps on disease spreading among the municipalities giving the mix patter observed for the dispersion of the disease: hierarchy + contiguity. We added this figure to answer one of the reviewers' comments that the observed pattern was trivial and expected, i.e., by the main railways. This figure is also crucial for figure 5 that contain the main airports involved in the disease transmission pattern. We added the following sentence in the section results and discussion (lines 220-225): "*People's movement is facilitated, and encouraged, due to transportation availability and commercial and social activities Likely SARS-CoV in 2003, the SARS-CoV-2 fastly spread among cities and countries due to airline network and ground transportation [22-24]. In the case of São Paulo State, the delay on closing the airports located at the inner municipalities probably contributed to the hierarchical dispersion of the disease on its territorial.*"

We added the following references:

[22] Litaker, J. R., Chou, J. Y., Novak, S., & Wilson, J. P. (2003). Implications of SARS: Medical geography and surveillance in disease detection. Annals of Pharmacotherapy,37(12), 1841-1848.

[23] Bowen Jr, J. T., & Laroe, C. (2006). Airline networks and the international diffusion of severe acute respiratory syndrome (SARS).Geographical Journal,172(2), 130-144.

[24] Ali, S. H., & Keil, R. (Eds.). (2011).Networked disease: Emerging infections in the global city (Vol. 44). John Wiley & Sons.

**3. What software (and what packages; if applicable) were used to model SEIR models. Include references. It is good practice to make the codes available. Or publish as a repository.**

It was implemented in language C using a Runge-Kutta method of 4^th^ order. However, the mathematical model was removed from the text, as suggested by Reviewer 1.

**4. The description has a major gap that need explanation how SEIR models are using the locomotion directionality information in the models. While the results discuss the flux network it has not been clearly explained in methods.**

The mathematical model was removed from the text, as suggested by Reviewer 1.

**5. SEIR modes: Basic model description is sufficient however, whether model simulations were run separately and simultaneously for each of the fourteen hotspot cities is unclear.**

They were run separately. However, the mathematical model was removed from the text, as suggested by Reviewer 1.

**6. Moreover, as the reviewer #1 has mentioned the models can be validated now that there is more data gathered.**

The mathematical model was removed from the text, as suggested by Reviewer 1.

**7. SEIR model assumptions and definitions: While SEIR models aren't new if you're assuming**

**- Random mixing vs specific contact rates among individuals in different age classes these need to be stated clearly**

The mathematical model was removed from the text, as suggested by Reviewer 1. Only to clarify, we were using a contact matrix to simulate the difference in the number of contact among individuals from different age classes.

**- Include the start day for each 14 cities in Table 2.**

Done.

**8. Lines 304 – 306. The hypothesis of hierarchy being more important than the contiguity to São**

**Paulo as a risk factor.**

We took out this sentence from the text since it was related to a discussion based on the mathematical model that was deleted from this new version as suggested by Reviewer 1.

**Results and discussion**

**1. The discussion needs a paragraph on limitations including: testing and data dependence, lack of validation, model assumptions, and the while the model helps to understand the early spread of COVID-19 with in a region, without having real-time data at more granular level, the difficulty to assess the risk profile or predict the risk spatiotemporally.**

We added two paragraphs in the Discussion section to describe the limitations of the study (lines 309-328): *"Limitations of the analysis include: (i) the no-identification of asymptomatic individuals and, potentially, mild or moderate infectious, since only symptomatic cases that seek for medical assistance have been tested; (ii) data dependence, i.e., data set does not distinguish between imported and autochthonous cases; (iii) the assumption that all individuals have the same degree of susceptibility and transmissibility of the disease, regardless the environment they live; (iv) the transmission is homogeneous within the cities; (v) mitigation strategies are the same everywhere. All those characteristics may variate according to the city because the number of tests that is distributed and performed among cities is not homogeneous; the number of contacts among people changes according to the city characteristics, such as the use of public transportation [29]; and people's adherence to social distancing really differed across the state, which may be related to the epidemics delay into reach the small inner cities, affecting people's risk perception [30].*

*Moreover, the data source in Brazil has been updated with some delay, regarding the occurrence of the infections [31]. Nevertheless, since the data used in this study is related to the arrival of infections in each city, which happened in early 2020, we expect the numbers to be trustful at the point of the analysis. Despite there is no data at a granular level, such as information about the address of the infection occurrence, the data is enough to perform the analysis and reach our goal, which was to study the spread of SARS-CoV-2 among cities.*"

The following references were added:

[29] Chen et al. 2020. Controlling urban traffic-one of the useful methods to ensure safety in Wuhan based on COVID-19 outbreak. doi: 10.1016/j.ssci.2020.104938

[30] Baicker et al 2020. Using social and behavioural science to support COVID-19 pandemic esponse. Nature Human Behaviour; p. 1--12.

[31] Do Prado et al (2020). Analysis of COVID-19 under-reporting in Brazil. Revista Brasileira de terapia intensiva,32(2), 224.

**2. The results or interpretations are not discussed in comparison to the recent literature on similar studies. Here are few examples. Authors may find more as there is a plethora of available literature:**

**- Chen et al. 2020. Controlling urban traffic-one of the useful methods to ensure safety in Wuhan based on COVID-19 outbreak. doi: 10.1016/j.ssci.2020.104938**

**- Bertuzzo et al., 2020. The geography of COVID-19 spread in Italy and implications for the relaxation of confinement measures. Doi: https://doi.org/10.1038/s41467-020-18050-2**

**How the information may be helpful for post COVID disease preparedness in cities:**

**- Pisano C. 2020. Strategies for Post-COVID Cities: AN insight to Paris en commun and Milano Sustainability 2020, 12(15), 5883; https://doi.org/10.3390/su12155883**

We thank the suggestion of the reviewer. To address this point, we added two paragraphs to the discussion section:

(lines 220-225): *"People's movement is facilitated, and encouraged, due to transportation availability and commercial and social activities Likely SARS-CoV in 2003, the SARS-CoV-2 fastly spread among cities and countries due to airline network and ground transportation [22-24]. In the case of São Paulo State, the delay on closing the airports located at the inner municipalities probably contributed to the hierarchical dispersion of the disease on its territorial."*

(lines 274-284): *"A relationship between disease spreading and territorial geography was also established in other epidemics [22,25]. Differently from São Paulo State, [26] showed that the first wave of SARS-CoV-2 pandemic in Germany followed a dispersion pattern called relocation diffusion process since the arrival of infections in Germany coincided with a traditional carnival festivity. Therefore, a single infected individual transmitted the infection to several others. After the festivities, people went back to their homeland, creating long-range connections, and new spots of infection spread, which were randomly distributed across the country. In São Paulo State, since all non-essential activity was limited, the spread followed the main routes of commercial relationships and supply distribution, in a hierarchical diffusion, firstly reaching the most important cities in São Paulo State, and locally spreading within their regions."*

And we added the new references:

[22] Litaker, J. R., Chou, J. Y., Novak, S., & Wilson, J. P. (2003). Implications of SARS: Medical geography and surveillance in disease detection.Annals of Pharmacotherapy,37(12), 1841-1848.

[23]Bowen Jr, J. T., & Laroe, C. (2006). Airline networks and the international diffusion of severe acute respiratory syndrome (SARS).Geographical Journal,172(2), 130-144.

[24]Ali, S. H., & Keil, R. (Eds.). (2011).Networked disease: Emerging infections in the global city (Vol. 44). John Wiley & Sons.

[25] Kuo, C. L., & Fukui, H. (2007). Geographical structures and the cholera epidemic in modern Japan: Fukushima prefecture in 1882 and 1895.International Journal of Health Geographics,6(1), 25.

[26]Kuebart, A., & Stabler, M. (2020). Infectious diseases as socio‐spatial processes: The Covid‐19 outbreak in Germany.Tijdschrift voor economische en sociale geografie,111(3), 482-496.

**Figures**

**Modifications of the figures have improved the overall understanding of the methods and results. However, here are specific comments on critical points:**

**1. Figure 1: The red circles represent 1, 100, and 5000 cases it seems. Please remove the period (.) from both legend and the map (Sao Paulo 9428). It is misleading**

Done.

**2. Figure 2 Insert a legend onto the figure: Color coded nodes and edges**

Done.

**3. Figure 4: The legend color scheme for "number of days since the first case" does not match what is on the map. Please use the same color scheme/ramp and edit the legend accordingly.**

Done.

**References**

**Several references have short-form of the journal name. Please edit the references according to Plos guidelines.**

Done.
